# High throughput sequencing of whole transcriptome and construct of ceRNA regulatory network in RD cells infected with enterovirus D68

**DOI:** 10.1186/s12985-021-01686-x

**Published:** 2021-11-07

**Authors:** Junzhuo Si, Xia Tang, Lei Xu, Huichao Fu, Huayi Li, Yonglin He, Jiajia Bao, Jialing Tang, Anlong Li, Nan Lu, Chun Yang

**Affiliations:** Department of Pathogenic Biology, College of Basic Medical Sciences, Chongqing Medical University, Chongqing, 400016 China

**Keywords:** Enterovirus D68, Whole transcriptome sequencing, Infection, Hub mRNAs, ceRNA regulatory network

## Abstract

**Background:**

With the advancement of sequencing technologies, a plethora of noncoding RNA (ncRNA) species have been widely discovered, including microRNAs (miRNAs), circular RNAs (circRNAs), and long ncRNAs (lncRNAs). However, the mechanism of these non-coding RNAs in diseases caused by enterovirus d68 (EV-D68) remains unclear. The goal of this research was to identify significantly altered circRNAs, lncRNAs, miRNAs, and mRNAs pathways in RD cells infected with EV-D68, analyze their target relationships, demonstrate the competing endogenous RNA (ceRNA) regulatory network, and evaluate their biological functions.

**Methods:**

The total RNAs were sequenced by high-throughput sequencing technology, and differentially expressed genes between control and infection groups were screened using bioinformatics method. We discovered the targeting relationship between three ncRNAs and mRNA using bioinformatics methods, and then built a ceRNA regulatory network centered on miRNA. The biological functions of differentially expressed mRNAs (DEmRNAs) were discovered through GO and KEGG enrichment analysis. Create a protein interaction network (PPI) to seek for hub mRNAs and learn more about protein–protein interactions. The relative expression was verified using RT-qPCR. The effects of Fos and ARRDC3 on virus replication were confirmed using RT-qPCR, virus titer (TCID_50_/ml), Western blotting.

**Results:**

375 lncRNAs (154 upregulated and 221 downregulated), 33 circRNAs (32 upregulated and 1 downregulated), 96 miRNAs (49 upregulated and 47 downregulated), and 239 mRNAs (135 upregulated and 104 downregulated) were identified as differently in infected group compare to no-infected group. A single lncRNA or circRNA can be connected with numerous miRNAs, which subsequently coregulate additional mRNAs, according to the ceRNA regulatory network. The majority of DEmRNAs were shown to be connected to DNA binding, transcription regulation by RNA polymerase II, transcription factor, MAPK signaling pathways, Hippo signal pathway, and apoptosis pathway, according to GO and KEGG pathway enrichment analysis. The hub mRNAs with EGR1, Fos and Jun as the core were screened through PPI interaction network. We preliminarily demonstrated that the Fos and ARRDC3 genes can suppress EV-D68 viral replication in order to further verify the results of full transcriptome sequencing.

**Conclusion:**

The results of whole transcriptome analysis after EV-D68 infection of RD cells were first reported in this study, and for the first time, a ceRNA regulation network containing miRNA at its center was established for the first time. The Fos and ARRDC3 genes were found to hinder viral in RD cells. This study establishes a novel insight host response during EV-D68 infection and further investigated potential drug targets.

**Supplementary Information:**

The online version contains supplementary material available at 10.1186/s12985-021-01686-x.

## Introduction

In 1962, EV-D68 was discovered in the throat swabs of four California infants suffering from pneumonia and bronchiolitis. Fermon, Franklin, Robison, and Rhyne are four distinct EV-D68 strains that have been isolated [1]. Case of EV-D68 infection were sporadic and rarely reported in the decades that followed, and it was regarded as a minor disease that went undiscovered. In the United States, an EV-D68 epidemic occurred in 2014 [2]. Retrospective research later revealed that Europe had a high rate of EV-D68 infection over the same period [3]. Since then, EV-D68 has attracted an increasing amount of interest.

MicroRNA (miRNA), long noncoding RNA (lncRNA), and circular RNA (circRNA) are types of noncoding RNA molecules revealed in recent decades that do not have the usual RNA function in the transcription and translation processes. MicroRNA (miRNA or miR) is an endogenous, non-coding, single-strand small RNA molecule with a length of 18-25 nucleotides [4]. LncRNA is an RNA molecule with a length of more than 200 bases [5], while circRNA is a type of closed-loop structure in RNA molecule, which is formed by special selective splicing of more than one exon [6]. Although lncRNA and circRNA are different in shape, they can compete with the same miRNA response element (MRE) to effectively control post-transcriptional transcription of miRNA and play a role similar to “sponge” [7, 8]. There are limited reports on the modulation of non-coding RNA during EV-D68 infection in human host cells at the moment. To thoroughly investigate the EV-D68 infection mechanism, we evaluated the entire transcriptional sequencing findings of EV-D68 infected RD cells and built a miRNA-centered ceRNA regulation network. Differential expression mRNAs (DEmRNAs), differential expression miRNAs (DEmiRNAs), differential expression lncRNAs (DElncRNAs), and differential expression circRNAs (DEcircRNAs) were screened using high-throughput sequencing of infection and control samples and the results of bioinformatics prediction. As a result, the ceRNA regulation network as well as the protein interaction network (PPI) were constructed further. GO and KEGG enrichment analyses were used to investigate the function of these DEmRNAs.

This is the first study to examine the whole transcriptome of EV-D68 infected RD cells and to develop the ceRNA regulation network and PPI in order to investigate the EV-D68 infection process.

## Materials and methods

### Cell culture, viral strain, virus infection and titration

RD cells were purchased from Beina Biotechnology Co. The RD cells were rapidly thawed and resuscitated from liquid nitrogen, and cultured in 10% fetal bovine serum (FBS; Hyclone, Thermo Fisher Scientific, Waltham, MA, USA) Dulbecco Modified Eagle Medium (DMEM; Hyclone, Thermo Fisher Scientific, Waltham, MA, USA) complete medium at 37 °C, 5% CO_2_ until the cell fusion degree reached 80–90%, subculture, or the next experiment.

The EV-D68 Fermon strain used in this study was preserved in the Chongqing Medical University laboratory of pathogenic biology and propagated in RD cells. In a nutshell, confluent cells were infected with EV-D68 at various multiplicities of infection (MOI), and viruses were collected from supernatants at the indicated infection time points. The tissue culture infectious dose (TCID_50_/ml) titers for EV-D68 were determined. The day before infection, RD cells were seeded in 96-well plates. The virus samples were serially diluted in DMEM with 2% FBS (10^−1^–10^−10^), and then each dilution was added to individual wells. For 2–5 days, the plates were incubated at 37 °C in 5% CO_2_. After 2 to 5 days, CPE was observed under a microscope. The REED-Munch endpoint calculation method was used to calculate virus titer, which was expressed as the 50% tissue culture infectious dose (TCID_50_/ml) [9].

### Cell transfection.

Plasmid’s transfection: RD cells were transfected with overexpression constructs using Lipo2000 (Invitrogen, carlsbadm, CA) at a 1:2  $\upmu$g:$\upmu$l ratio of plasmid to transfection reagent on the basis of the manufacturer’s protocol. siRNA’s transfection: Using Lipo2000 (Invitrogen, carlsbadm, CA) with 50 nM siRNA and 2 μl transfection reagents, siRNA was transfected into RD cells, according to the method of the manufacturer.

### RNA extraction

Total RNA was extracted from the cells using TRIzol (Invitrogen, Carlsbad, CA, USA) according to manual instruction. In brief, TRIzol was added to the cells and incubated for 5 min at 37 °C before being transferred to new centrifuge tubes. A one-fifth volume of chloroform was added, and samples were agitated and allowed to stand at room temperature for 5 min before being centrifuged at 12,000*g* for 15 min at 4 °C. The supernatants were collected, and the RNA pellets were washed in 1 ml 75% ethanol, then centrifuged for 5 min at 12,000*g* at 4 °C. The pellets were then air-dried and resuspended in DEPC-treated H_2_O and stored at − 80 °C until further use. Subsequently, total RNA was qualified and quantified using a Nano Drop and Agilent 2100 bioanalyzer (Thermo Fisher Scientific, MA, USA); all RNA samples with RIN $\ge$ 8 and 28 s/18 s $\ge$ 1 was considered.

### High throughput sequencing and construction of RNA library

#### mRNA, LncRNA, circRNA library construction and sequencing

DNase I was used to degrade double-stranded and single-stranded DNA in total RNA, after which the reaction products were purified using magnetic beads, and the rRNA was removed using RNase H or the Ribo-Zero method (human, mouse, plants) (Illumina, USA). The purified mRNA from the previous steps was fragmented into small pieces using fragment buffer at the proper temperature. Then, using the First Strand Reaction System, first-strand cDNA was created, as well as second-strand cDNA. Magnetic beads were used to purify the reaction result, and then A-Tailing Mix and RNA Index Adapters were added and incubated to carry out end repair. PCR was used to amplify the cDNA fragments with adapters, and Ampure XP Beads were used to purify the results. For quality control, the library was validated on an Agilent Technologies 2100 bioanalyzer. The splint oligo sequence heated denatured and circularized the double-stranded PCR products above. The final library was formatted using single strand circular DNA (ssCir DNA). The final library was amplified with phi29 (Thermo Fisher Scientific, MA, USA) to produce DNA nanoballs (DNBs) with over 300 copies of one molecule. DNBs were inserted into the patterned nanoarray, and pair end 100 base readings were generated on the BGISEQ500 platform (BGI-Shenzhen, China). In the sequencing process, the methods for determining mRNAs, lncRNAs, and circRNAs are detailed below. For lncRNAs and mRNAs: Clean reads were obtained and stored in FASTQ format after the sequencing data was filtered with SOAPnuke (v1.5.2). HISAT2 was used to map the clean reads to the reference genome (v2.0.4). Then, to detect fusion genes and differential splicing genes (DSGs), Ericscript (v0.5.5) and rMATS (V3.2.5) were utilized. Bowtie2 (v2.2.5) was used to align clean reads to the gene set, a database created by BGI (Beijing Genomic Institute in ShenZhen) that comprised known and new, coding and noncoding transcripts, and then RSEM was used to calculate gene expression levels (v1.2.12). For circRNAs: circRNA is predicted by CIRI and Find_circ software. After merging the results of the two softwares, quantitative and differential expression analysis of circRNA was performed. (1) CIRI identifies the circRNA according to the reads at the circRNA connection point. (2) Find_circ identifiles circRNA by looking for reverse variable shear joint sequence (back-spliced junction). For a more detailed description of the process, see Additional file 1.

#### Small RNA library construction and sequencing

To make the library, start with 1μg total RNA from each sample. First, the short RNA fragment of 18-30nt was isolated and retrieved using electrophoresis. Second, the 18–30 nt small RNAs were ligated to adenylated 3' adapters annealed to unique molecular identifiers (UMI) before being ligated to 5' adapters. To transcribe and synthesize cDNA, use SuperScript II Reverse Transcriptase (Invitrogen, USA). PCR amplification yielded a large number of cDNA fragments. QIAquick Gel Extraction Kit (QIAGEN, Valencia, CA) was used to purify cDNA extracted from an agarose gel containing a 110-130bp target fragment. Finally, an Agilent 2100 biological analyzer (Thermo Fisher Scientific, MA, USA) was used to assess the library's quality. Sequencing is done using the BGISEQ-500 platform (BGI-Shenzhen, China). Identification method for miRNAs, after we get the raw sequencing data and filter it to get a clean reads, Bowtie2 (2 2.2.9) was used to map the clean tags to the reference genome and other sRNA databases such as miRbase, siRNA, piRNA, and snoRNA. Cmsearch (1.1.2) was used for Rfam mapping. Piano was used to predict piRNAs and miRDeep2 (2.0.0.8) was used to predict novel miRNAs by looking at the secondary structure. For a more detailed description of the process, see Additional file 1.

### Analysis of differentially expressed genens

In order to evaluate the expression of transcripts, according to the length of fragments and the reading counts mapped to these fragments, the transcripts per thousand bases and per million mapped reading fragments (FPKM) were calculated by using Cufflinks software (v2.1.1.). In general, FPKM≥0.1 indicates that the transcript is expressed. According to the experimental design, we used the software of cufflinks to screen the differentially expressed genes (DEGs) between the EV-D68 infected group and the control group. We set |Log_2_(Fold change) |≥ 1 and Q.value<0.05 as the index to identify the significant difference between infection group and non-infection group.

### The construction of the ceRNA regulatory network

Based on the ceRNA theory, we constructed a ceRNA regulatory network DEcircRNAs, DElncRNAs, DEmiRNAs and DEmRNAs to demonstrate the regulatory relationship among circRNA, lncRNA, miRNA and mRNA. We use miRanda (http://www.microrna.org/microrna) to predict the target genes of miRNA. The ceRNA network which consisted of lncRNA-miRNA pairs, circRNA-miRNA pairs, and miRNA-mRNA pairs with the same miRNA nodes was visualized by Cytoscape 3.8.2.

### Functional analysis

In order to further understand the infection mechanism of EV-D68, we conducted GO analysis and KEGG signaling pathway analysis to predict the potential function of DEmRNAs. In the GO analysis, we analyze the following three aspects: biological processes (BP), cellular components (CC) and molecular functions (MF). KEGG signaling pathway analysis is to understand the functional classification of differentially expressed genes involved in the pathway or exercise (KEGG as a reference resource for gene and protein annotation, http://www.genome.jp/kegg/), and carry out statistical analysis, with P value < 0.05 as the standard for functional enrichment analysis.

### The establishment of the PPI network and identification of hub mRNAs

The String (http://sting-db.org) online database could construct PPI network, which was visualized by Cytoscape (v3.8.2). Next, using the degree algorithm in the cytohubba plug-in of Cytoscape, the degree value could be calculated and thus the hub genes could also be screened from PPI network.

### Quantitative real-time polymerase chain reaction (RT-qPCR)

The Mir-X miRNA First-Strand synthesis kit (TaKaRa, China) was used on total RNA for reverse transcription of miRNA according to the manufacturer's instructions. The PrimeScript™ RT reagent Kit with gDNA Eraser (TaKaRa, China) was used on total RNA for reverse transcription of mRNA according to the manufacturer's instructions. The mRQ 3′ Primer supplied with the kit is the 3′primer for miRNA RT-qPCR. The Oligo 7 software was used to design specific primers according to the sequence of the selected genes, and the specificity of the primers was evaluated by NCBI Primer-BLAST and dissolution curve. The qualified primers were produced by Tsingke Biotechnology Company (Beijing). The miRNAs expression level was normalized to U6, and the expression level of mRNAs were normalized to $\beta$-actin. The data analysis was carried out by using 2^−ΔΔCt^ method. The primers for RT-qPCR were shown in Table 1.

**Table 1:** Primer sequences for a quantitative real‐time polymerase chain reaction

Name	Sequence (5′ → 3′)
ARRDC3-F	AGCAGCATTTTGTTACTGACT
ARRDC3-R	ACTTTTGTGTATGTCCCGTTT
Fos-F	CCGGGGATAGCCTCTCTTACT
Fos-R	CCAGGTCCGTGCAGAAGTC
EGR1-F	AAGAAAAGCCAAGCAAACCAA
EGR1-R	AACGGAACAACACTCTGACAC
Jun-F	TCCAAGTGCCGAAAAAGGAAG
Jun-R	CGAGTTCTGAGCTTTCAAGGT
hsa-miR-411-3p-F	UAUGUAACACGGUCCACUAACC
hsa-let-7c-3p-F	CUGUACAACCUUCUAGCUUUCC
hsa-miR-30c-1-3p-F	CUGGGAGAGGGUUGUUUACUCC
hsa-miR-323a-5p-F	AGGUGGUCCGUGGCGCGUUCGC
hsa-miR-320b-F	AAAAGCUGGGUUGAGAGGGCAA
hsa-miR-532-3p-F	CCUCCCACACCCAAGGCUUGCA
U6-F	CTCGCTTCGGCAGCACATATACT
U6-R	ACGCTTCACGAATTTGCGTGTC
$\beta$-actin-F	CATTGCCGACAGGATGCAG
$\beta$-actin-R	CGGAGTACTTGCGCTCAGGA

### Western blot assay

The total protein extraction kit is used to extract total protein from the treated cells according to the manufacturer's instructions (Thermo Fisher Scientific, Waltham, MA, USA). Protein BCA Assay Kit was used to determine the protein concentration in the lysate (Beyotime, Shanghai, China). The protein was separated on a 10% sodium dodecyl sulfate polyacrylamide gel electrophoresis and then transferred to a PVDF membrane for western blot detection. Block the membrane for 1 hour with 5% skim milk in Tris-buffer saltwater containing Tween 20 at room temperature. They were then incubated with primary antibodies against EV-D68 VP1 (1:5000, GeneTex, Shanghai, China), Fos (1:5000, Proteintech Group, Wuhan, China), ARRDC3 (1:5000, Proteintech Group, Wuhan, China), β-actin (1:8000, Proteintech Group, Wuhan, China), and GAPDH (1:8000, Proteintech Group, Wuhan, China) overnight at 4°C, before being incubated for 1 hour at room temperature with secondary antibodies bound to horseradish peroxidase. The target protein is seen and quantified with Bio-hypersensitive Rad's ECL chemiluminescence kit (New Cell & Molecular Biotech Co., Ltd., Suzhou, China).

### Plasmid construction and siRNA synthesis

To construct the overexpression plasmids for Fos and ARRDC3, we used Table 2 primers to amplify the coding regions of Fos and ARRDC3 from the genomes of RD cells (synthesized by Tsingke Biotechnology Beijing Co., Ltd.). PCDH-CMV-MCS-EF1-copGFP-T2A-Puro (CD513B) was digested with ECORI and XhoI, and the PCR fragment was cloned into the necessary location to generate Fos and ARRDC3 overexpression plasmids (Additional file 2). Tsingke Biotechnology Beijing Co., Ltd. produced siRNA (Table 3).

**Table 2:** Primer sequences used in PCR

Name	Sequence (5′ → 3′)
Eco-ARRDC3-F	aagGAATTCGCCACCATGATGTTCTCGGGCTTCAAC
Xho-ARRDC3-myc-R	aaCTCGAGTTACAGATCCTCTTCTGAGATGAGTTTTTGTTCCAGGGCCAGCAGCGTGGG
Eco-Fos-F	aaaGAATTCGCCACCATGATGTTCTCGGGCTTCAAC
Xho -Fos-myc-R	ggCTCGAGTTACAGATCCTCTTCTGAGATGAGTTTTTGTTCCAGGGCCAGCAGCGTGGG

**Table 3:** ARRDC3 and *c-fos* siRNA sequence

Name	Sequence (5′ → 3′)
human-ARRDC3-siRNA-1-F	CUUAUCAUCUGGAAAGACATT
human-ARRDC3-siRNA-1-R	UGUCUUUCCAGAUGAUAAGTT
human-ARRDC3-siRNA-2-F	GGUAUAUGUGGAUAUUCCUTT
human-ARRDC3-siRNA-2-R	AGGAAUAUCCACAUAUACCTT
human-ARRDC3-siRNA-3-F	CACUGUUUGCAUAUAUCCATT
human-ARRDC3-siRNA-3-R	UGGAUAUAUGCAAACAGUGTT
human-Fos -siRNA-1-F	GGAGACAGACCAACUAGAATT
human-Fos-siRNA-1-R	UUCUAGUUGGUCUGUCUCCTT
human-Fos-siRNA-2-F	GCAUGGAGCUGAAGACCGATT
human-Fos-siRNA-2-R	UCGGUCUUCAGCUCCAUGCTT
human-Fos-siRNA-3-F	GUUAUCUCCAGAAGAAGAATT
human-Fos-siRNA-3-R human-siRNA-NC-F human-siRNA-NC-R	UUCUUCUUCUGGAGAUAACTT UUCUCCGAACGUGUCACGUTT ACGUGACACGUUCGGAGAATT

### Statistical analysis

The statistical analysis was performed by using the GraphPad Prism Software Version 8.0 (Grapad Prism Software, La Jolla, CA). All data in the study were represented as mean $\pm$ standard deviation from at least three independent experiments. Statistical comparisons were made by unpaired Student’s t test (for two-group comparisons) or one-way analysis of variance (for multiple group comparisons). P $<0.05$ was considered statistically significant.

## Results

### Construction cell model in vitro of EV-D68

**Fig. 1:**
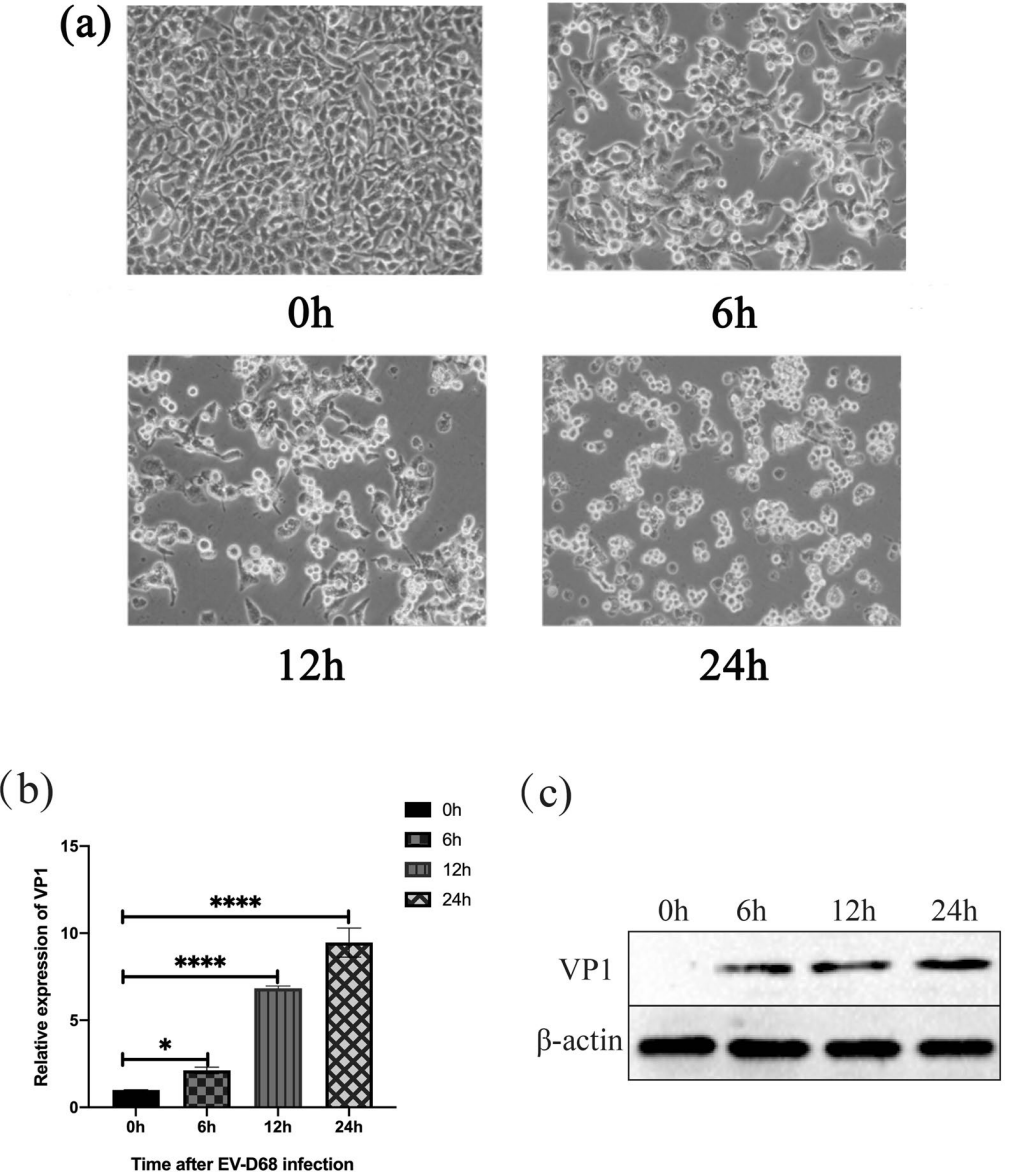
EV-D68 Construction Cell Model in Vitro. **a** 0, 6, 12, 24 h after infection with MOI = 1. The results revealed that CPE was present in the cells. **b** RT-qPCR was used to evaluate the relative expression of viral VP1 in RD cells after infection with EV-D68 at a MOI of 1 for 6, 12, and 24 h, respectively. The relative expression of viral VP1 increased as the time gradient increased. **c** Western blot results revealed that the expression of viral VP1 increased steadily over time at the protein level. *p < 0.05, ****p < 0.0001. *EV-D68* Enterovirus D68, *CPE* cytopathic effect

We examined the cytopathic effect (CPE) and viral VP1 expression in RD cells to ensure that they are an efficient cell model for researching the molecular mechanism of EV-D68 infection. CPE can be noticed under the condition of MOI=1 infection for 0, 6, 12, 24 h, as shown in Fig. 1a. It can be seen from the Fig. 1a that RD cells obviously shrink, float, and even die as time goes on after being infected with EV-D68. Second, under the circumstance of MOI=1, we apply RT-qPCR and western blot to detect viral VP1 expression both on RNA level (Fig. 1b) and protein level (Fig. 1c) at different time gradients. After RD cells were infected with EV-D68, the expression of VP1 at the RNA and protein levels increased in a time dependent manner. In conclusion, RD cells are an effective cell model for investigating the process of EV-D68 infection.

### Overview of whole transcriptome analysis

Before data analysis, low quality readings, linker contamination and high content of unknown base N are removed from the original sequencing to ensure the quality of the sequencing data. After processing, we obtained the following data. The three uninfected groups produced 21379210, 20001268, and 22052381 clear reads in the small RNA library. Clean reads were produced by three infection groups: 21809488, 23711936, and 23881547, respectively. Similarly, three uninfected groups produced 1052700, 1015600, and 1086900 clean reads in the lncRNA library. Three infection groups produced clean reads of 1114000, 1128200, and 1128100, respectively. These data support the accuracy of the subsequent analysis process.

### Differential expression analysis

**Fig. 2:**
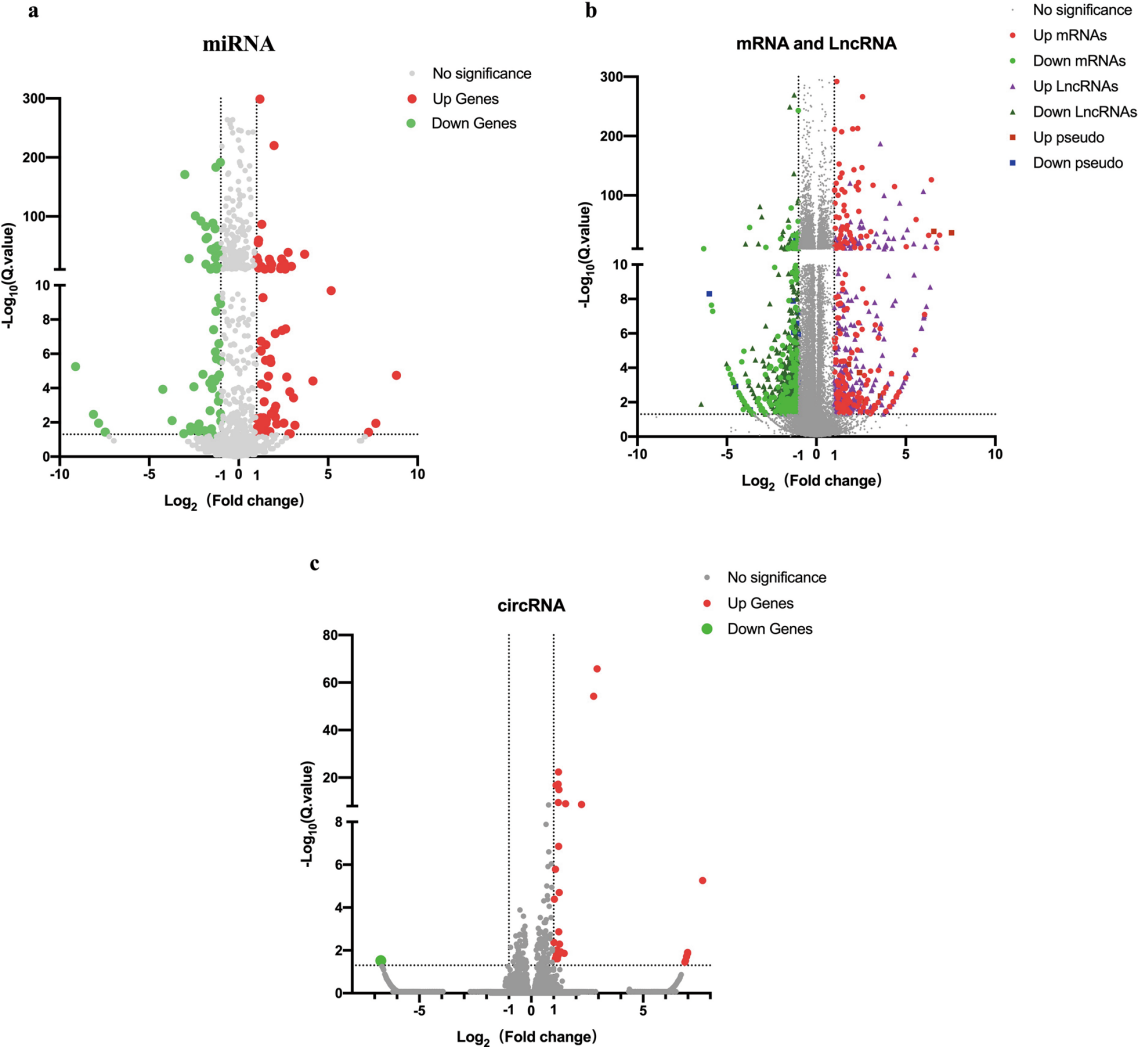
volcano map of differentially expressed genes. The horizontal and vertical axes in the figure represent Log_2_(Fold change) and -Log_10_(Q.Value) respectively. We used |Log_2_(Fold change) |≥ 1, Q.Value < 0.05 as cut-off criteria and determined it’s significance

**Fig. 3:**
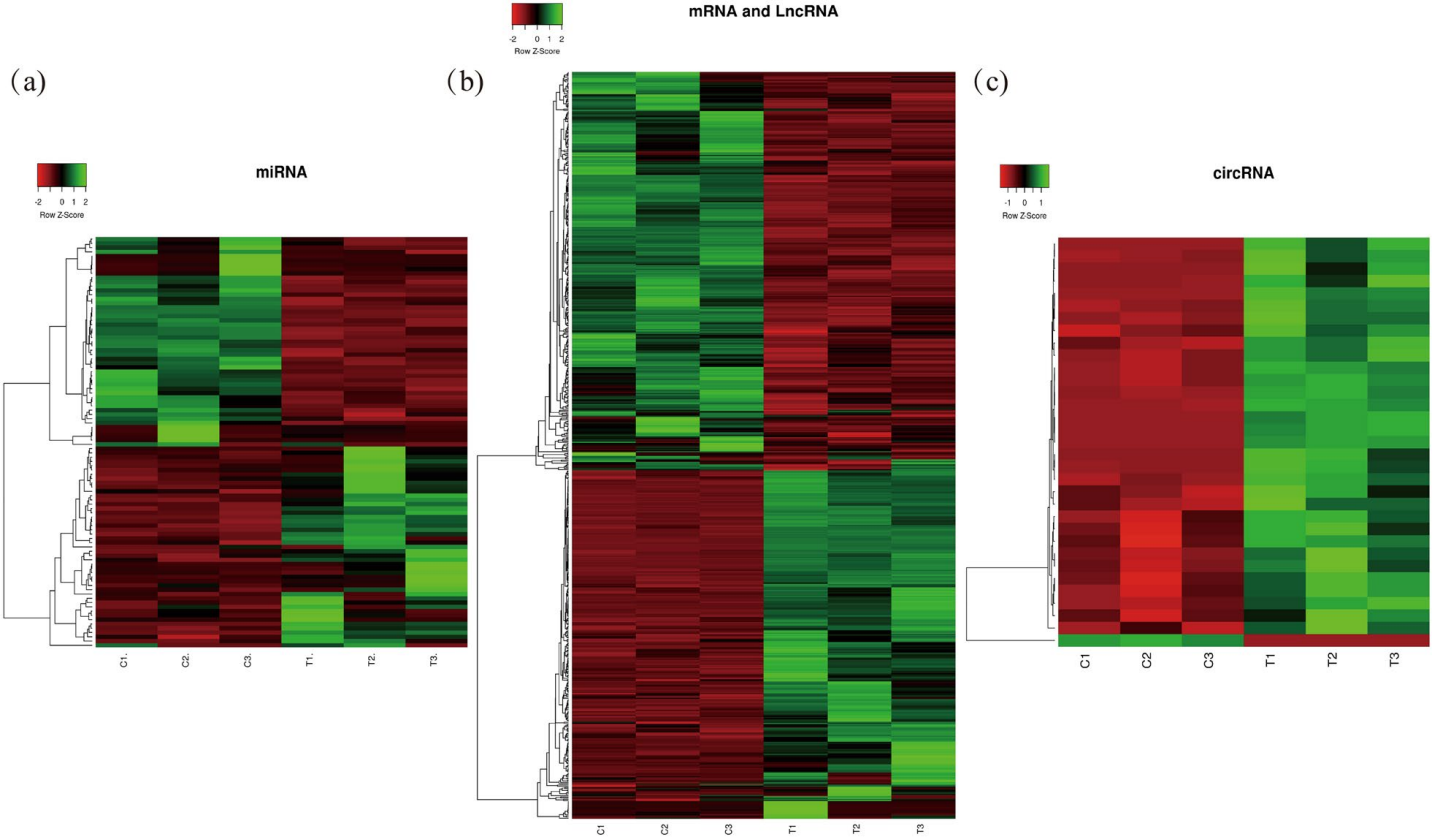
Cluster analysis of differentially expressed genes heat map. Heat maps of miRNA differently expressed genes (**a**), mRNA and lncRNA differentially expressed genes (**b**), and circRNA differentially expressed genes (**c**) were displayed. Each column of the control and experimental groups is represented by the horizontal axis. The color represented the quantity of expression of a sample in each group. The hue progressively changed from green to crimson as the amount of expression increased

Because differentially expressed genes will be utilized as the basis for further analysis in high-throughput whole transcriptome sequencing, screening differentially expressed genes is critical. We screened the entire dataset using the following criteria: |Log_2_(Fold changel) |$\ge 1$, Q.value < 0.05. We can see that there are significant differences in the expression profiles of mRNAs (Fig. 2b), miRNAs (Fig. 2a), lncRNAs (Fig. 2b), and circRNAs (Fig. 2c) after analyzing the data to create the volcano map. We also created a cluster analysis heat map, which depicted the expression of each gene in control and infected groups of mRNAs (Fig. 3b), miRNAs (Fig. 3a), lncRNAs (Fig. 3b), and circRNAs (Fig. 3c) in great detail.375 lncRNAs (154 upregulated, 221 downregulated), 33 circRNAs (32 upregulated, 1 downregulated), 96 miRNAs (49 upregulated, 47 downregulated), and 239 mRNAs (135 upregulated, 104 downregulated) were screened and identified as differentially expressed genes in this sequencing analysis. The whole information of differentially expressed genes is shown in Additional file 3–6.

### GO and KEGG pathway analysis

**Fig. 4:**
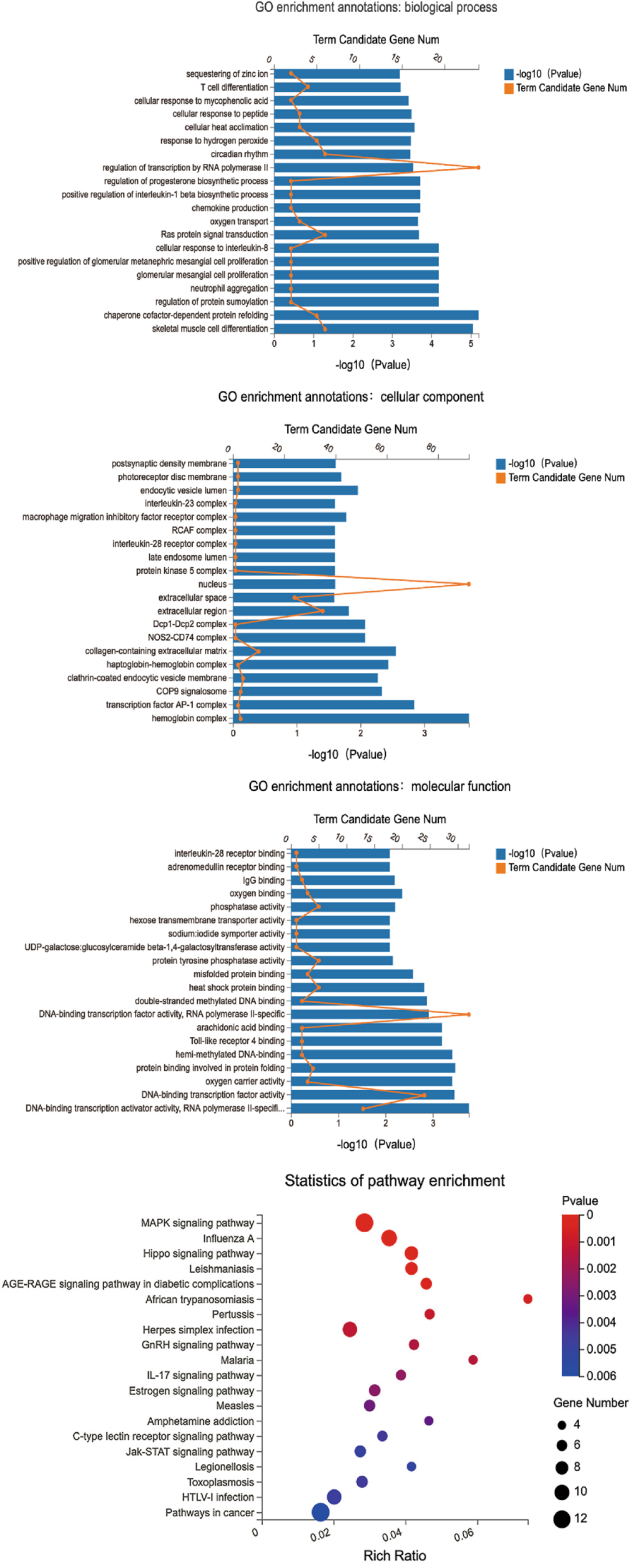
GO enrichment highlights include biological process, cell composition, molecular function, and the KEGG signaling pathway enrichment bubble graphic, from top to bottom. The size of − Log_10_(P.Value) in the GO enrichment map is indicated by the blue bar, and the number of genes enriched in each GO annotation is indicated by the yellow line. In the KEGG signal pathway enrichment bubble diagram, the size of the bubble reflects the number of genes enriched in each signal pathway, and the color of the spots reflects its significance level

To define the function of DEmRNAs in various aspects, GO enrichment analysis was conducted from three perspectives: molecular function, biological process, and cellular components. Simultaneously, we utilize KEGG signal pathway analysis to better understand which signal pathway DEmRNAs are involved in. The infection mechanism of EV-D68 will be described in further depth using these two ways. Figure 4 depicts the results, which are analyzed from the perspectives of biological process (BP), cellular components (CC), and molecular function (MF). “RNA polymerase II regulation of transcription”, “protein signal transduction”, “hydrogen peroxide reaction”, “nucleus”, “extracellular domain and extracellular space”, “DNA binding transcription factor activity”, and “RNA polymerase II specificity” are the most common types in BP, CC, and MF, respectively. Signaling pathway is mainly enriched in "MAPK signaling pathway", "Hippo signaling pathway", "leishmaniasis", "AGE-RAGE signaling pathway of diabetic complications" and "apoptosis signaling pathway" according to our KEGG pathway analysis. Table 4 lists the genes that are enriched in the top 20 signaling pathways. We have summarized the detailed data of GO enrichment and KEGG signal pathway analysis, as shown in Additional files 7–8. According to the results, RNA polymerase II transcriptional regulation, DNA binding transcription factor activity and MAPK signal pathway are at the forefront of all results, so our follow-up research will focus more on the genes enriched on them.

**Table 4:** Enriched genes of the top20 KEGG signaling pathways

KEGG terms	Input genes
MAPK signaling pathway	PLA2G4B, GADD45G, DUSP1, FOS, HSPA1A, HSPA1B, HSPA6, JUN, KITLG, GADD45B, PTPN7, BGIG9606_54754
Influenza A	INSL6, HLA-DRA, HSPA1A, HSPA1B, HSPA6, DNAJB1, IL12A, JUN, OAS1, RSAD2
Hippo signaling pathway	RASSF1, CTGF, ID1, ID2, SMAD7, SERPINE1, FRMD1, FZD9
Leishmaniasis	INSL6, LOC112268237, FOS, HLA-DRA, IL12A, JUN, FCGR2C
AGE-RAGE signaling pathway in diabetic complications	INSL6, EGR1, JUN, SERPINE1, PIM1, BGIG9606_41843
African trypanosomiasis	HBA1, HBA2, HBB, IL12A
Pertussis	TMED-TICAM2, FOS, IL12A, JUN, IL23A
Herpes simplex infection	INSL6, FOS, HLA-DRA, IL12A, JUN, OAS1, CFP, CD74, BGIG9606_54498
GnRH signaling pathway	PLA2G4B, HBEGF, EGR1, JUN, BGIG9606_41843
Malaria	HBA1, HBA2, HBB, IL12A
IL-17 signaling pathway	FOS, FOSB, JUN, S100A8, S100A9
Estrogen signaling pathway	HBEGF, FOS, HSPA1A, HSPA1B, HSPA6, JUN
Measles	INSL6, HSPA1A, HSPA1B, HSPA6, IL12A, OAS1
Amphetamine addiction	ARC, FOS, FOSB, JUN
C-type lectin receptor signaling pathway	EGR2, EGR3, IL12A, JUN, IL23A
Jak-STAT signaling pathway	INSL6, IFNL1, IL7R, IL12A, IL23A, PIM1
Legionellosis	HSPA1A, HSPA1B, HSPA6, IL12A
Toxoplasmosis	INSL6, HLA-DRA, HSPA1A, HSPA1B, HSPA6, IL12A
HTLV-I infection	EGR1, EGR2, FOS, HLA-DRA, JUN, ATF3, ZFP36, FZD9, BGIG9606_41843
Pathways in cancer	GADD45G, INSL6, RASSF1, FOS, IL7R, IL12A, JUN, KITLG, GADD45B, IL23A, PIM1, FZD9

### Identification of hub mRNAs from the PPI network

**Fig. 5:**
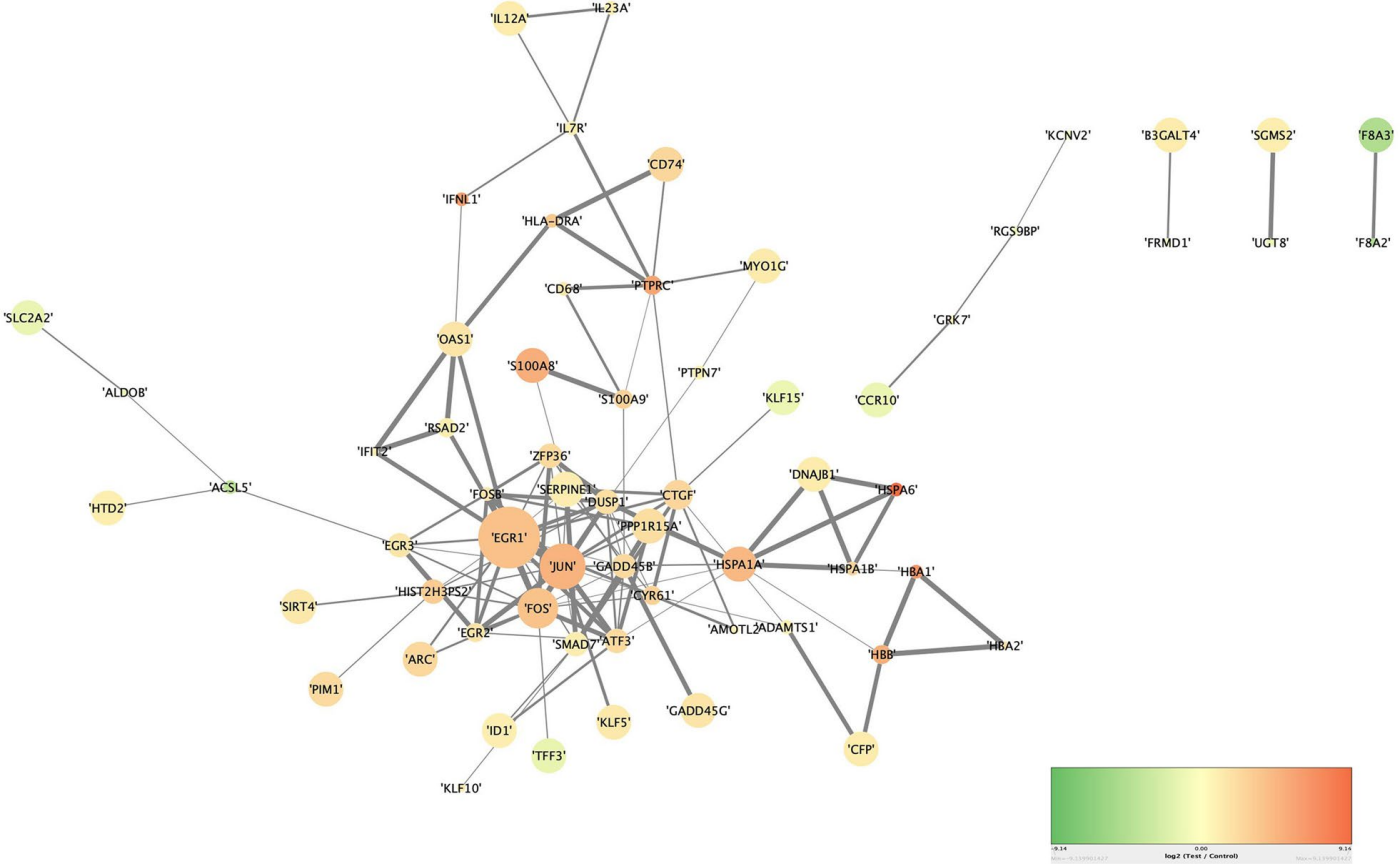
PPI interaction network. The color of the node changes from green to red according to Log_2_(Fold change). The frequency of each mRNA in the network determines the size of each node. Furthermore, the total score of nearby genes went from fine to coarse

Protein-protein interaction networks are made up of proteins that interact with one another to help with biological signaling, gene expression regulation, energy and material metabolism, cell cycle regulation, and other life activities. Understanding the molecular mechanism of EV-D68 infection necessitates a systematic examination of protein interactions. Based on the biological interactions of 239 DEmRNAs, a PPI network with 64 nodes and 130 edges was constructed to further identify their associations at the protein level (Fig. 5).

**Fig. 6:**
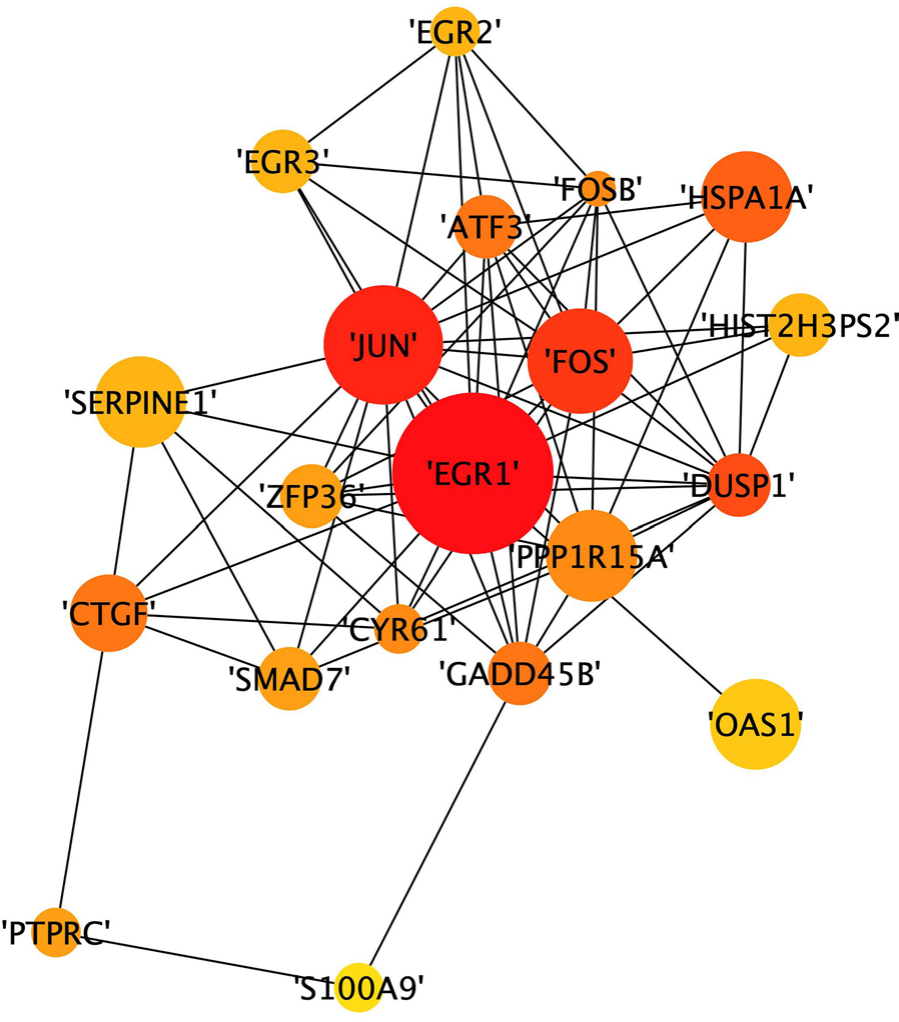
interaction of hub mRNAs. The top20 hub mRNAs were chosen using the cytohubba software's degree algorithm. The degree score of each mRNA in the PPI interaction network determines the size and color of the nodes

By screening Hub mRNAs, we can see more clearly, and intuitively which genes are important. The PPI network was then visualized in Cytoscape (version 3.8.2) and analyzed by using 'Degree method' in plugin Cytohubba to identify the network's top 20 hub mRNAs (Fig. 6). The major genes in hub mRNA include EGR1, Fos, Jun, and PPP1R15A, as shown in the diagram, implying that these genes may play a key part in the mechanism of EV-D68 infection.

### Construction of the ceRNA regulatory network

**Fig. 7:**
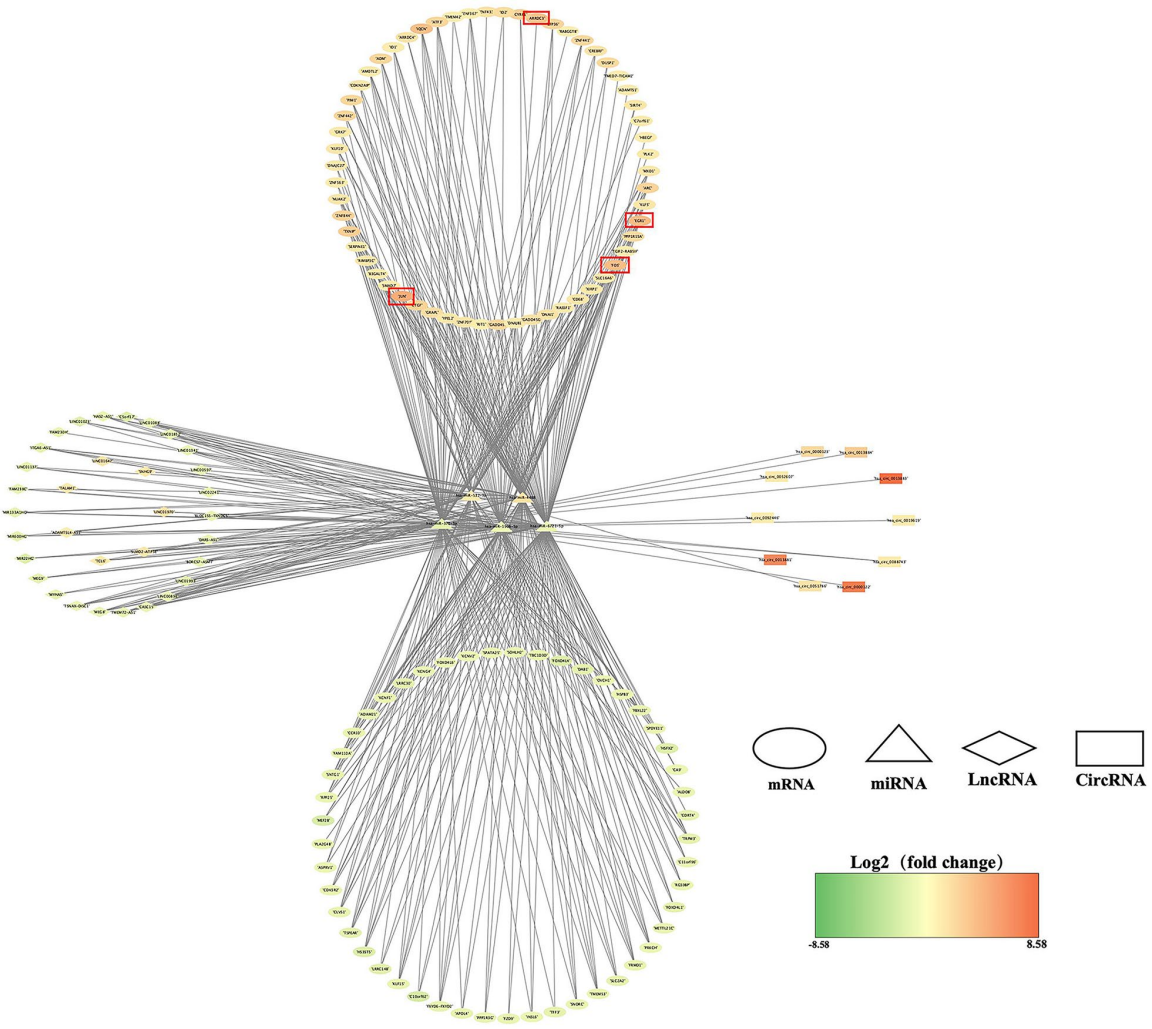
The ceRNA regulatory network centered on the top5 miRNAs. The ellipse, triangle, diamond, and rectangle in this diagram represent mRNA, miRNA, lncRNA, and circRNA, respectively. The color of the node changes from green to red according to Log_2_(Fold change). Hub mRNAs are shown by the red rectangle in the image

The ceRNA hypothesis, published by the Pier Paolo Pandolif research group in 2011, is a model of gene expression regulation, suggesting that mRNA can regulate each other via miRNA response elements (MREs) [10]. The proposal of ceRNA hypothesis endows mRNA and non-coding RNA with more extensive biological functions. Because of the building of the ceRNA regulatory network, we can better understand the significance of the interaction between non-coding RNA and mRNA in the process of EV-D68 infection. 120 mRNAs, 60 miRNAs, 40 lncRNAs, and 33 circRNAs were chosen from the differentially expressed genes to participate in the creation of the ceRNA regulation network. We chose the top 5 miRNAs to showcase because the full ceRNA regulation network map is too big (Fig. 7). The complete ceRNA regulatory network diagram is shown in Additional file 9.

In the Fig. 7, we also mark 4 hub mRNAs with a red box, and these hub mRNAs will be targeted by miRNA to perform their functions. According to the findings, several lncRNAs and circRNAs can target miRNA at the same time, and numerous mRNAs can be regulated at the same time. This information will allow us to locate the genes that play a significant part in the EV-D68 infection process more quickly.

### RT-qPCR analysis

**Fig. 8:**
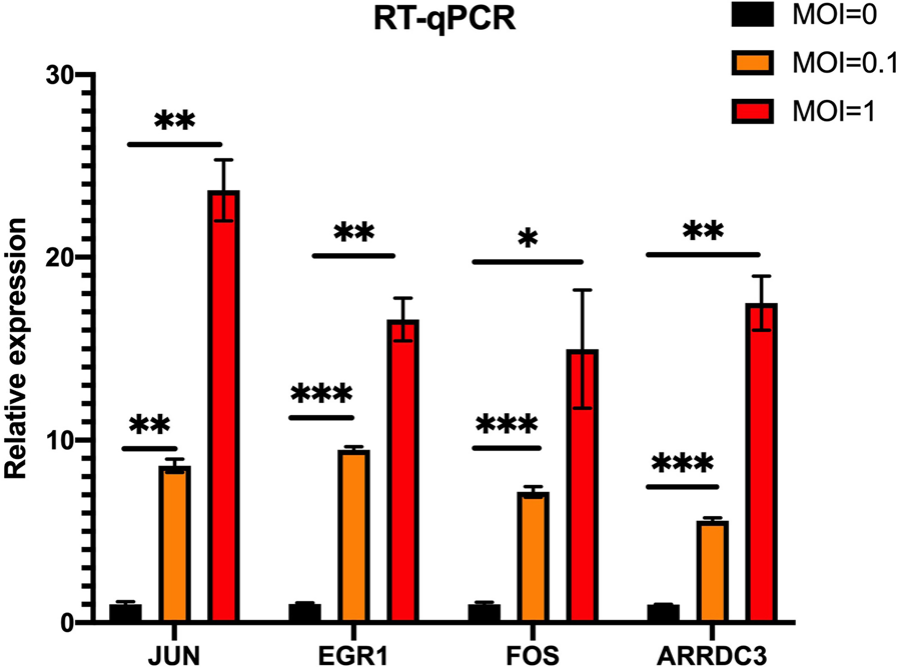
DEmRNA RT-qPCR validation results. The names of genes and their relative expression are represented by the horizontal and vertical axes, respectively. The black square represents the MOI = 0 control group, whereas the yellow and red squares represent the MOI = 0.1 and MOI = 1 infection group, respectively. *p < 0.05, **p < 0.01, ***p < 0.001

To ensure the accuracy of the sequencing results, we carried out RT-qPCR verification. 4 DEmRNAs (including 3 hub genes) and 6 DEmiRNAs were chosen for RT-qPCR validation of sequencing expression results. The expression trend of four DEmRNAs gradually increased after RD cells were infected with MOI 0, 0.1, and 1 for 24 hours, as shown in Fig. 8.

**Fig. 9:**
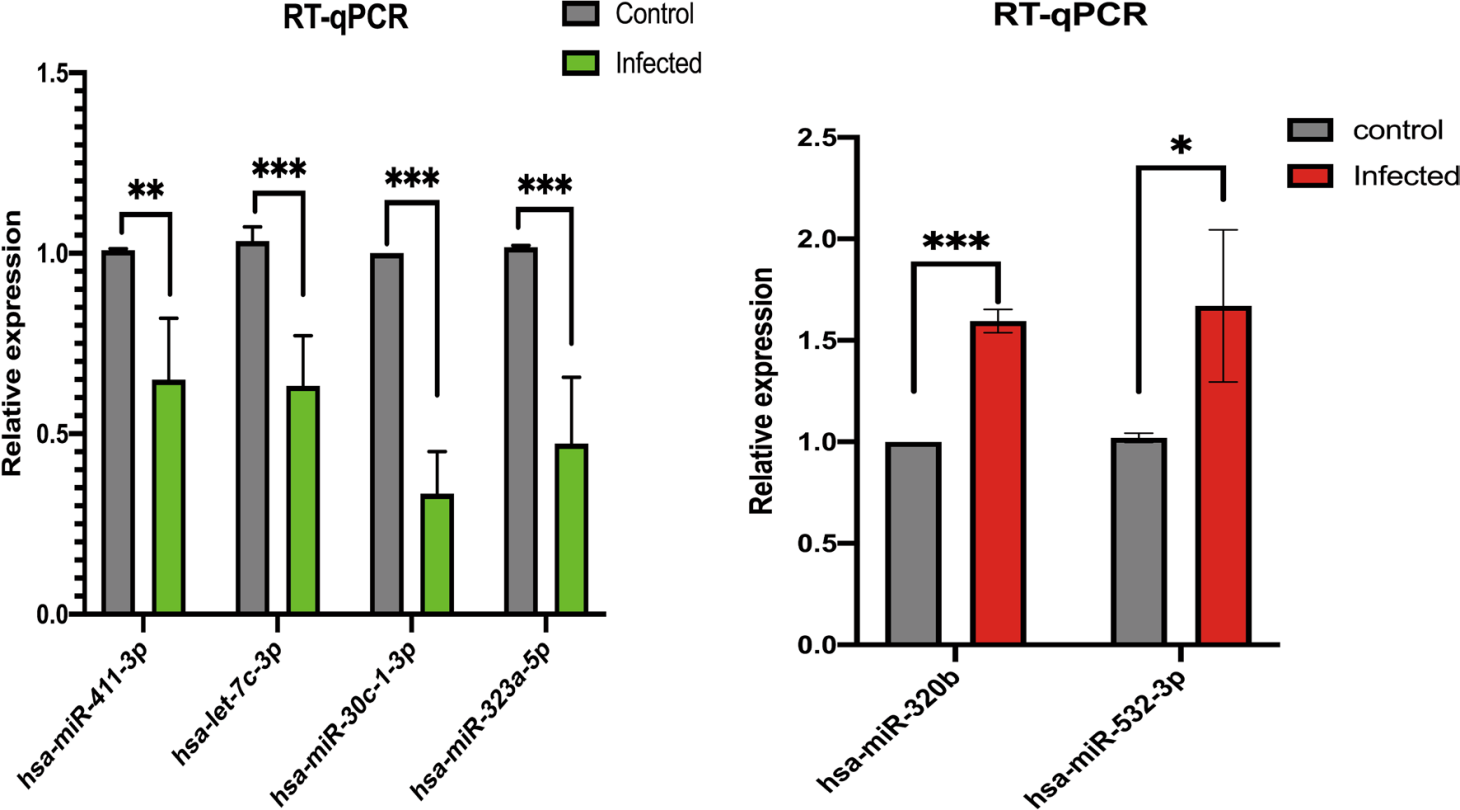
verification results of DEmiRNAs RT-qPCR. The names of genes and their relative expression are represented by the horizontal and vertical axes, respectively. Under the infection condition of MOI = 1, the gray column represents the MOI = 0 control group, while the green and red columns indicate the down-regulated gene and up-regulated gene experimental groups, respectively. *p < 0.05, **p < 0.01, ***p < 0.001

Figure 9 indicated a downregulation trend of 4 DEmiRNAs and an upregulation trend of 2 DEmiRNAs 24 hours after we infected RD cells with MOI=1.

Compare our verification results with our sequencing data. The expression trend of the verified genes was basically consistent with the results of sequencing data. These results can ensure the accuracy of our sequencing results and provide a guarantee for follow-up experiments.

### Fos and ARRDC3 can inhibit EV-D68 replication

As mentioned above, we found that Fos and ARRDC3 genes were up-regulated in different degrees after EV-D68 infected RD cells. However, because the effect of these two genes on viral replication has not been investigated, we created vectors for Fos, ARRDC3 and empty vector (CD513B-Fos, CD513B-ARRDC3, CD513B-NC) and synthesized their siRNA (siRNA-Fos, siRNA-ARRDC3, siRNA-NC) to overexpress and knockdown these two genes.

**Fig. 10:**
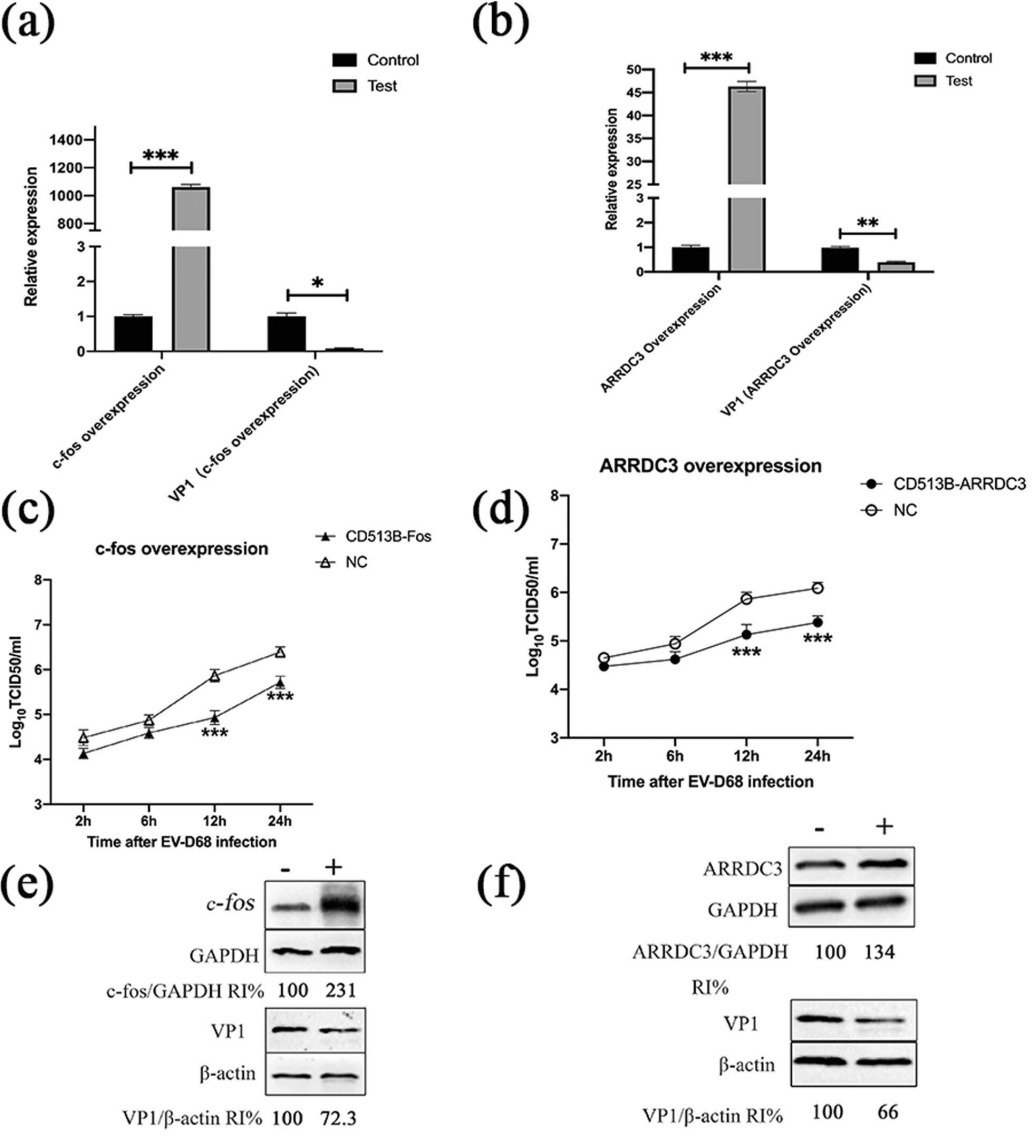
Effects of overexpression of Fos and ARRDC3 on EV-D68 replication. CD513B-Fos (CD513B-NC) or CD513B-ARRDC3 (CD513B-NC) were transfected into RD cells, which were then infected with EV-D68 at a multiplicity of infection (MOI) of 1 after 48 h. RT-qPCR was used to evaluate the quantities of EV-D68 viral VP1 RNA in the Fos overexpression group (**a**) and the ARRDC3 overexpression group (**b**). At the indicated time points after infection, virus titers in culture supernatants from the Fos overexpression group (**c**) and the ARRDC3 overexpression group (**d**) were measured. The protein expression of VP1 in Fos overexpression group (**e**) and ARRDC3 overexpression group (**f**) were determined by western blot assay. *p < 0.05, **p < 0.01, ***p < 0.001

In order to observe whether they affect virus replication in three aspects: RNA, protein, and virus titer. The overexpression vector (CD513B-Fos or CD513B-ARRDC3) or empty vector (CD513B-NC) were transiently transfected into RD cells to investigate the effect of Fos or ARRDC3 overexpression on EV-D68 replication. RT-qPCR results 48 hours after transfection revealed that the overexpression vector significantly increased the expression of Fos or ARRDC3 in RD cells as compared to the control group (Fig. 10a, b). RD cells were also infected with EV-D68 at MOI=1 for 20 hours post transient transfection. In comparison to the control group, overexpression of Fos or ARRDC3 significantly suppressed EV-D68 replication, according to viral titer results (Fig. 10c, d). In the same way, we utilized RT-qPCR to identify viral VP1 expression in RD cells. The primary component of the EV-D68 capsid is VP1 [11, 12]. After overexpression of Fos or ARRDC3, the viral VP1 gene was significantly suppressed compared with the control group (Fig. 10a, b). The viral VP1 protein was shown to be significantly reduced after overexpression of Fos or ARRDC3 at the protein level (Fig. 10e, f). It is suggested that overexpression of Fos or ARRDC3 can suppress the replication of EV-D68 virus in RD cells.

**Fig. 11:**
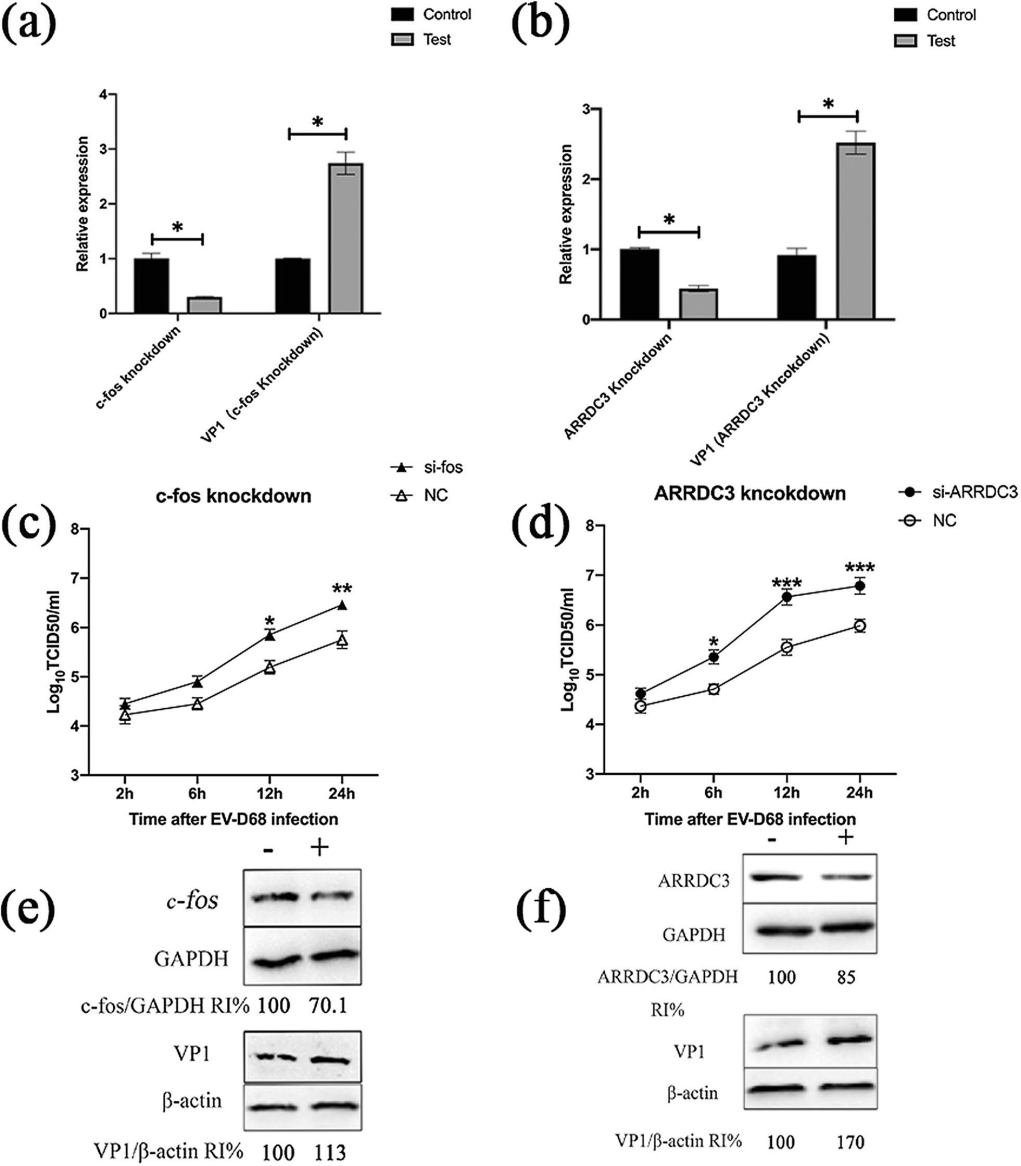
Effects of knockdown of Fos and ARRDC3 on EV-D68 replication. siRNA-Fos (siRNA-NC) or siRNA-ARRDC3 (siRNA-NC) were transfected into RD cells, which were then infected with EV-D68 at a multiplicity of infection (MOI) of 1 after 48 h. RT-qPCR was used to evaluate the quantities of EV-D68 viral VP1 RNA in the Fos knockdown group (**a**) and the ARRDC3 knockdown group (**b**). At the indicated time points after infection, virus titers in culture supernatants from the Fos knockdown group (**c**) and the ARRDC3 knockdown group (**d**) were measured. The protein expression of VP1 in Fos knockdown group (**e**) and ARRDC3 knockdown group (**f**) were determined by western blot assay. *p < 0.05, **p < 0.01, ***p < 0.001

In RD cells, transient transfection of two genes' siRNA (siRNA-Fos, siRNA-ARRDC3) could suppress the expression of two genes, respectively. The expression of Fos or ARRDC3 in RD cells transfected with siRNA was significantly decreased 48 hours after transfection, compared to the siRNA-NC group (Fig. 11a, b). RD cells were infected with EV-D68 for 20 hours under the condition of MOI=1 when 48 hours of siRNA or siRNA-NC transfection. The replication of EV-D68 was significantly increased after knockdown of Fos or ARRDC3 compared to the control group, according to virus titer findings (Fig. 11c, d). The results of RT-qPCR showed that the knockdown of Fos or ARRDC3 could promote the increase of the EV-D68 VP1 gene (Fig. 11a, b). Western blot analysis further confirmed that the expression of VP1 protein in RD cells with Fos or ARRDC3 knockdown was higher than that in the control group (Fig. 11e, f). It is suggested that Fos or ARRDC3 knockdown can promote the replication of EV-D68 virus in RD cells.

As a result, we determined that after EV-D68 invaded RD cells, the Fos and ARRDC3 genes were increased, inhibiting EV-D68 viral replication.

## Discussion

Many research results have discovered that non-coding RNA is considered a kind of "noise" in the mechanism of virus infection and plays a key function as a result of the advancement of molecular biology and bioinformatics [13]. Recent research has discovered that differential expression of lncRNA in enterovirus 71 (EV71) infected host cells can eventually lead to apoptosis. In 2019, Liao et al. discovered that EV71 infection inhibited the endogenous expression of lnc-irak3-3, allowing miR-891b to be released from the chelation process of lnc-irak3-3. The proportional increase in miR-891b would further suppress the expression of mRNA GADD45β, resulting in a reduction in EV71-induced apoptosis [14]. Cheng et al. discovered that EV-D68 could stimulate the accumulation of markers such as Ras GTPase activated protein binding protein 1 (G3BP1), T cell antigen 1 (TIA1), and human antigen R (HuR) in the early stages of infection, but prevented their accumulation later on. Infection with EV-D68 caused HuR, TiA1, and G3BP1 to colocalize, resulting in the formation of a typical SG dependent on protein kinase R (PKR) and eIF2 phosphorylation, implying that TiA1, HuR, and G3BP1 could target EV-D68's 3' untranslated region (UTR), inhibiting viral replication. These findings suggest that EV-D68 infection is mediated by ncRNAs and mRNA [15].

The competing endogenous RNAs (ceRNA) hypothesis reveals a novel RNA interaction mechanism. We know that miRNA can silence genes by binding to mRNA, whereas ceRNA can regulate gene expression by binding to miRNA competitively. CeRNA binds to miRNA via microRNA response elements (MREs), causing miRNA failure, indicating that a ncRNA- miRNA regulatory pathway exists [10]. Xu et al. discovered in 2019 that long noncoding RNAs (lncRNAs) play a key role in hepatocellular carcinoma (HCC) caused by hepatitis B virus (HBV) infection. They discovered two important ceRNA pathways in the pathogenesis by constructing the ceRNA regulatory network: FAM138B—hsa-miR-30c—CCNE2/RRM2 and SSTR5-AS1—hsa-miR-15b-5p—CA2 [16]. However, there are no complete sequencing results of EV-D68 infected host cells or ncRNA-mediated regulatory network of enterovirus infection mechanism in the existing reports.

We used EV-D68 to conduct high-throughput sequencing of RD cells after infection in order to explore the whole transcriptome and biological function of ncRNAs following EV-D68 infection in host cells, from which we conducted bioinformatics mining to offer a solid foundation for the mechanism investigation.

In our study, we first sequenced three control samples and three infected samples to analyze the DEGs. The results showed that there were 239 DEmRNAs, 96 DEmiRNAs, 375 DElncRNAs and 33 DEcircRNAs. To further explore their roles after infection, we constructed the interaction network of circRNA, lncRNA, miRNA and mRNA. This network showed miRNAs can be targeted by multiple circRNAs and lncRNAs simultaneously, and then they jointly regulate more mRNAs. As a result, the creation of the ceRNA regulatory network provides a solid foundation for the research of prospective EV-D68 drug targets as well as the virus's infection process. For example, in our analysis, we found that hsa-circ- 0051786 and LINC00597 can be associated with hsa-miR-532-3p at the same time, and ultimately regulate the gene expression of Fos and EGR1. In our ceRNA regulatory network, we discovered that XIXT (lncRNA) can bind to hsa-miR-150-5p to control Fos expression. The XIST/miR-150-5p/Fos pathway has also been shown to impact sepsis-induced myocardial damage [17].

GO and KEGG signal pathway analysis were used to discover the biological roles and pathways of DEmRNAs. Gene expression regulation, cell cycle, and apoptosis are all essential enrichment routes, according to our findings. The MAPK signaling route and the Hippo signaling pathway are the two most important pathways. The MAPK signaling pathway, also known as mitogen activated protein kinase, has been discovered to have a key role in gene regulation and cytoplasmic function [18]. Shi et al. found that when enterovirus 71 (EV71) infected RD cells, MAPK signal molecules were highly up-regulated, as were the expression of AKT2, ELK1, Jun, Fos, and other associated genes to variable degrees. They suggest the differential expression of MAPK signaling molecules in EV71-infected RD cells is linked to host cell death and inflammatory cytokine release [19]. Through the combined reaction of kinases, the Hippo signaling pathway can stimulate cell proliferation and decrease the production of apoptotic genes, achieving the goal of regulating cell proliferation, apoptosis, and stem cell self-renewal [20]. If the Hippo pathway is disrupted, cancer is likely to occur. Hu et al. found elevated levels of blood fibrinogen (PLG) in HBV positive liver cancer tissues and cells in 2021. They discovered that suppressing HBV replication caused PLG to enhance apoptosis in HBV-HCC cells in vitro. SRC, PLG's downstream target gene, was substantially expressed in HCC caused by HBV and was closely connected to the Hippo signaling pathway, according to their GO and KEGG study. As a result, they suggest that PLG supports the role of the Hippo signaling pathway in the survival of HBV-HCC cells by up-regulating and activating the expression of SRC, hence boosting HBV-induced HCC progression [21]. Fos, Jun, and GADD45B are the key genes enriched in the MAPK signaling pathway in our study. RASSF1, ID1, and FRMD1 et al. are the main genes enriched in the Hippo signal pathway. The Hippo signal pathway, for example, is rich in RASSF1. They deduce that miR-602/RASSF1A may be an early diagnostic sign mediated by HBV because it has been documented that miR-602 can play a cancer-promoting function in the incidence of HBV-related liver cancer through consistent RASSF1A [22]. So, do these genes have a part in the process of EV-D68 invading RD cells in viruses or host cells? As a result, we'll focus our follow-up research on relevant genes enriched in the MAPK and Hippo signal pathways.

In addition, we constructed a PPI network and filtered 20 hub mRNAs from it in order to better understand the mechanism of the ceRNA regulating network. Virus replication and cell apoptosis are strongly linked by the proteins Fos, Jun, and EGR1. Fos is a class of nuclear protein transcription factors that regulate cell development, division, proliferation, differentiation, and even programmed cell death. Its expression has drawn the attention of numerous academics since it has an impact on a variety of living activities and processes [23]. Jun was the first carcinogenic transcription factor to be found. It has the ability to encode a protein that functions similarly to a viral protein in that it can bind directly with particular target DNA to regulate gene expression [24]. Fos is a crucial regulator in the transmission of HCV, according to Kang et al. They initially silenced and overexpressed the Fos gene, finding that overexpression dramatically boosted HCV transmission in the cell model, but silence resulted in a significant decrease in HCV transmission. They next employed a Luciferase report and an immunofluorescence assay to confirm their hypothesis. Finally, they came to the conclusion that the Fos gene was critical in the propagation of HCV [25]. Yuan et al. found that Fos gene also played a key role in the study of neuronal apoptosis. They concluded that potassium deficiency induced neuronal apoptosis was mediated by up regulating JUN/ATF2 heterodimer and down regulating Fos expression. All these indicate that Fos gene and Jun gene play an important role in the process of apoptosis [26]. In our experiment, we found that Fos and Jun genes are enriched in MAPK signaling pathway. The abnormality of MAPK signaling pathway mentioned above will directly affect the host cell apoptosis and inflammatory cytokine secretion. Despite the fact that ARRDC3 is not among the first 20 hub mRNAs screened in the PPI network, it has been reported that knocking down ARRDC3 in Hela cells results in a significant reduction in cell growth and sensitivity to HPV16 pseudovirion infection, indicating that ARRDC3 is involved in the process of HPV infection into cells [27].

According to the previous description, Fos also has a role to play in the virus infection process. As a result, we devised an experiment in which we overexpressed and knocked down Fos and ARRDC3 in RD cells to investigate what influence they had on EV-D68. It was discovered that the Fos and ARRDC3 genes could suppress the replication of EV-D68 based on RNA level, viral titer, and protein level tests.

In follow-up experiments, we will use the discoveries of prior bioinformatics predictions to delve deeper into the targeting mechanisms of ncRNAs and mRNAs.

## Conclusion

To summarize, after EV-D68 infected RD cells, whole transcriptional sequencing analysis was performed for the first time, and a miRNA-centered regulatory network was constructed to investigate the putative infection mechanism of EV-D68. The MAPK signal pathway and the Hippo signal pathway have been discovered. In addition, using the PPI network, hub-mRNAs (EGR1, Fos, Jun, and so on) were discovered. Some hub-mRNAs and miRNAs were verified by RT-qPCR to verify the results of whole transcriptome sequencing. Fos and ARRDC3 were verified at the protein level, RNA level and virus titer preliminarily prove that the two genes could suppress EV-D68 replication. Our findings add to the body of evidence supporting a more in-depth investigation of the mechanism of EV-D68 infection

## Supplementary Information

**Additional file 1**. How mRNAs, miRNAs, lncRNAs and circRNAs are determined during sequencing

**Additional file 2**. Plasmid map and sequencing results of CD513B-ARRDC3 and CD513B-Fos

**Additional file 3**. The whole of differentially expression genes of mRNAs

**Additional file 4**. The whole of differentially expression genes of miRNAs

**Additional file 5**. The whole of differentially expression genes of lncRNAs

**Additional file 6**. The whole of differentially expression genes of circRNAs

**Additional file 7**. GO annotation of DEGs in RD cell

**Additional file 8**. KEGG enrichment of DEGs in RD cell

**Additional file 9**. The full ceRNA regulatory network. The ellipse, triangle, diamond, and rectangle in this diagram represent mRNA, miRNA, LncRNA, and circRNA, respectively. According to the Log_2_(fold change) of RNAs, the node color changes gradually from green to red in ascending order

## Data Availability

All data and materials in this manuscript are fully available without restriction.

## Abbreviations

EV-D68: Enterovirus D68
RDcells: Rhabdomyosarcoma Cell
ncRNAs: Non- coding RNAs
miRNA: Micro RNA
lncRNA: Long non-coding RNA
circRNA: Circular RNA
DEG: Differentially expression gene
DEmRNAs: Differentially expression mRNAs
DEmiRNA: Differentially expression micro RNA
DElncRNA: Differentially expression long non-coding RNA
DEcircRNA: Differentially expression circular RNA
ceRNA: Competing endogenous RNA
PPI: Protein–protein interaction
MRE: MiRNA response element
