# Supplementary material for: High throughput sequencing of whole transcriptome and construct of ceRNA regulatory network in RD cells infected with enterovirus D68
Source: Virol J. 2021 Nov 7;18:216. doi: 10.1186/s12985-021-01686-x (PMC8574037; doi:10.1186/s12985-021-01686-x)
Supplement: Supplementary file 1 — Additional file 1. How mRNAs, miRNAs, lncRNAs and circRNAs are determined during sequencing [file 12985_2021_1686_MOESM1_ESM.pdf]

How mRNAs, miRNAs, lncRNAs and circRNAs are determined during sequencing.

For miRNAs:

Raw tags refer to the raw sequencing data. The raw tags were treated as follows: low quality tags were removed, tags with 5 primer contaminants were removed, tags without 3 primer contaminants were removed, tags were removed without insertion, tags with poly A were removed, and tags shorter than 18 nt were removed. After filtering, Bowtie2 was used to map the clean tags to the reference genome and other sRNA databases such as miRbase, siRNA, piRNA, and snoRNA. cmsearch was used for Rfam mapping. Piano was used to predict piRNAs and miRDeep2 was used to predict novel miRNAs by looking at the secondary structure.

For lncRNAs and mRNAs:

Clean reads were obtained and stored in FASTQ format after the sequencing data was filtered with SOAPnuke (v1.5.2) by (1) removing reads containing sequencing adapter; (2) removing reads whose low-quality base ratio (base quality less than or equal to 5) is more than 20%; (3) removing reads whose unknown base ('N' base) ratio is more than 5%. HISAT2 was used to map the clean reads to the reference genome (v2.0.4). Then, to detect fusion genes and differential splicing genes (DSGs), Ericscript (v0.5.5) and rMATS (V3.2.5) were utilized. Bowtie2 (v2.2.5) was used to align clean reads to the gene set, a database created by BGI (Beijing Genomic Institute in ShenZhen) that comprised known and new, coding and noncoding transcripts, and then RSEM was used to calculate gene expression levels (v1.2.12).

For circRNAs:

circRNA is predicted by CIRI and Find_circ software. After merging the results of the two softwares, quantitative and differential expression analysis of circRNA was performed.

1. CIRI identifies the circRNA according to the reads at the circRNA connection point, and the comparison of the reads at the connection point is very special. CIRI recognizes circRNA according to three models, in which the read at the join point is called junction read.

Model 1: circRNA is formed by cyclization of three exons. Due to the restriction of sequence reading, junction read only covers part of the sequence of the starting exon and the terminating exon. The alignment position of these two parts of reads is opposite to that of the genome.

Model 2: circRNA is formed by cyclization of three exons. Because the length of one exon at the junction is too short, junction read not only covers the two parts of the starting exon and the terminating exon, but also covers the partial sequence of one exon in the middle.

Model 3: circRNA is formed by cyclization of one exon. Junction read not only covers the whole exon, but also reads part of the sequence repeatedly.

2. Find_circ identifiles circRNA by looking for reverse variable shear joint sequence (back-spliced junction).

The approximate steps are as follows: First, the fragments from unmapped to the reference genome are obtained, and the 20mers at both ends of each fragment is compared to the cut exon, the anchor position arranged in the opposite direction is picked, and the sequence between the alignment is extended, and the GU/AG is the cutting site.
