# Supplementary figures and images for: High throughput sequencing of whole transcriptome and construct of ceRNA regulatory network in RD cells infected with enterovirus D68
Source: Virol J. 2021 Nov 7;18:216. doi: 10.1186/s12985-021-01686-x (PMC8574037; doi:10.1186/s12985-021-01686-x)

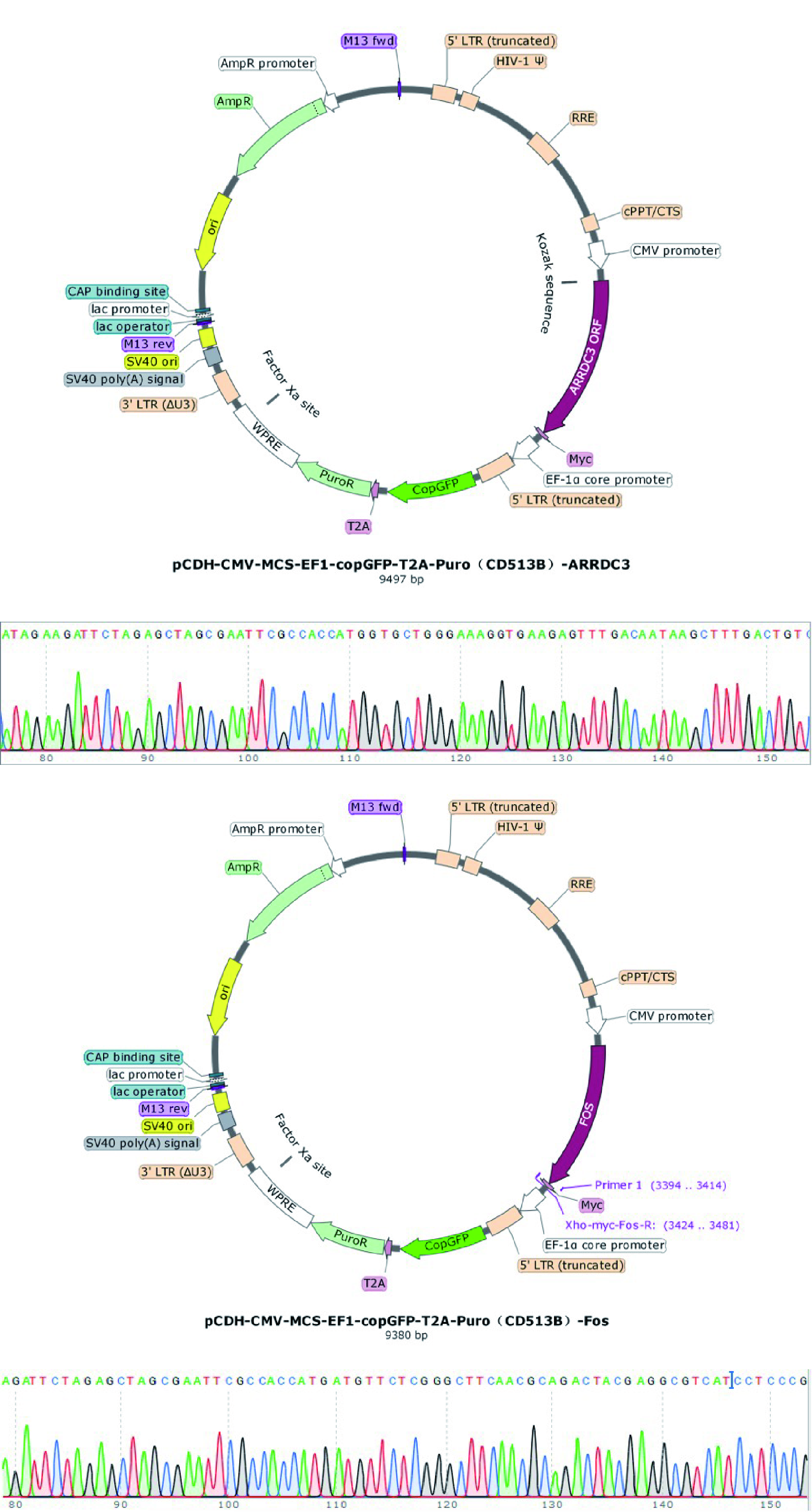

Supplement: Supplementary file 2 — Additional file 2. Plasmid map and sequencing results of CD513B-ARRDC3 and CD513B-Fos [file 12985_2021_1686_MOESM2_ESM.jpg]

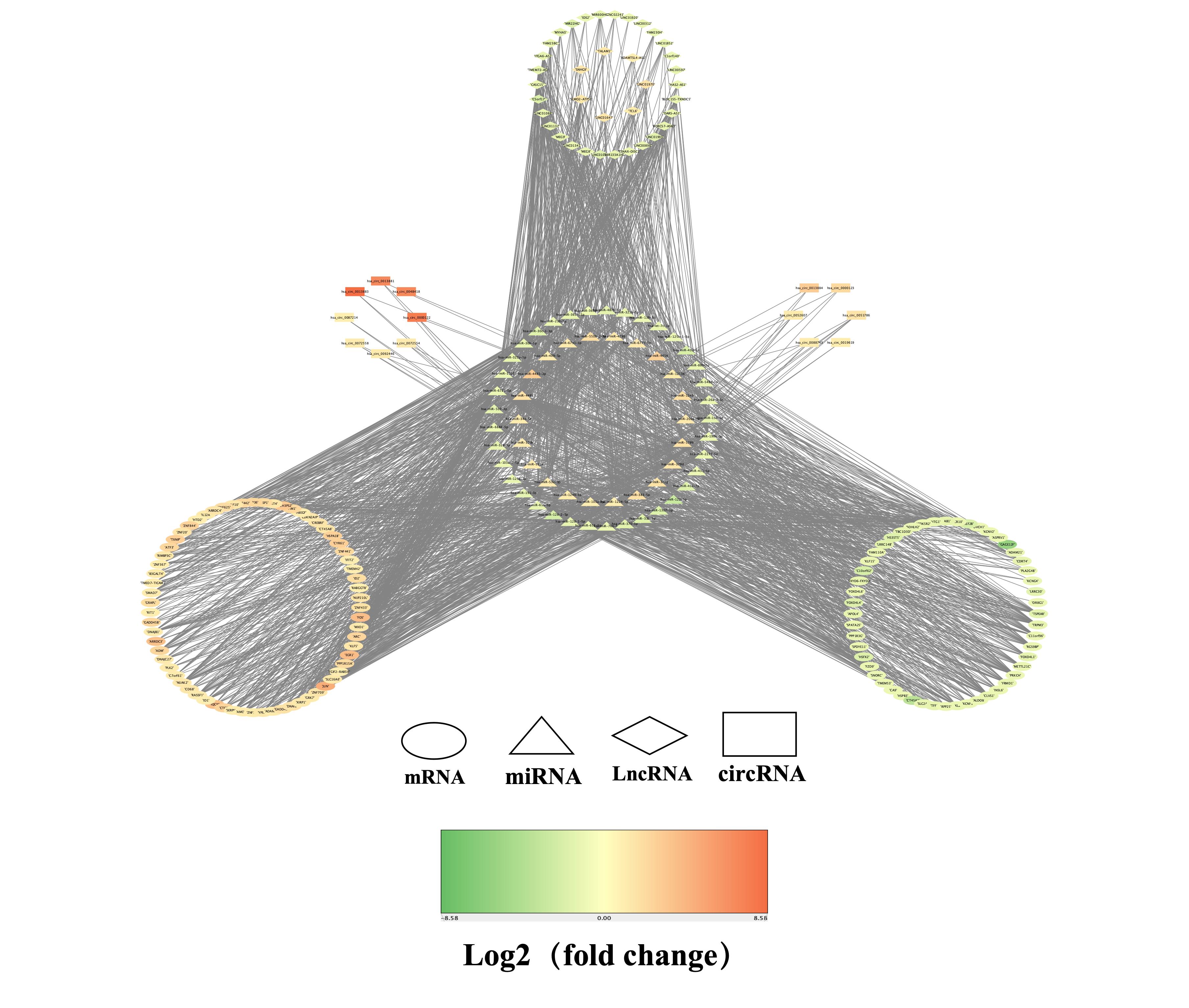

Supplement: Supplementary file 9 — Additional file 9. The full ceRNA regulatory network. The ellipse, triangle, diamond, and rectangle in this diagram represent mRNA, miRNA, LncRNA, and circRNA, respectively. According to the Log2(fold change) of RNAs, the node color changes gradually from green to red in ascending order [file 12985_2021_1686_MOESM9_ESM.jpeg]
